# Facial Width-To-Height Ratio (fWHR) Is Not Associated with Adolescent Testosterone Levels

**DOI:** 10.1371/journal.pone.0153083

**Published:** 2016-04-14

**Authors:** Carolyn R. Hodges-Simeon, Katherine N. Hanson Sobraske, Theodore Samore, Michael Gurven, Steven J. C. Gaulin

**Affiliations:** 1 Department of Anthropology, Boston University, Boston, Massachusetts, United States of America; 2 Department of Anthropology, University of California Santa Barbara, Santa Barbara, California, United States of America; Universidad de Chile, CHILE

## Abstract

Facial width-to-height ratio (fWHR) has been proposed as a sexually dimorphic signal in humans that develops under the influence of pubertal testosterone (T); however, no studies have examined the association between fWHR and T during the phase in which facial growth is canalized—adolescence. In a sample of adolescent Tsimane males, we evaluate the relationship between T, known T-derived traits (i.e. strength and voice pitch), and craniofacial measurements. If fWHR variation derives from T’s effect on craniofacial growth during adolescence, several predictions should be supported: 1) fWHR should increase with age as T increases, 2) fWHR should reflect adolescent T (rather than adult T per se), 3) fWHR should exhibit velocity changes during adolescence in parallel with the pubertal spurt in T, 4) fWHR should correlate with T after controlling for age and other potential confounds, and 5) fWHR should show strong associations with other T-derived traits. Only prediction 4 was observed. Additionally, we examined three alternative facial masculinity ratios: facial width/lower face height, cheekbone prominence, and facial width/full face height. In contrast to fWHR, all three alternative measures show a strong age-related trend and are associated with both T and T-dependent traits. Overall, our results question the status of fWHR as a sexually-selected signal of pubertal T and T-linked traits.

## Introduction

Animals have evolved mechanisms to detect, decode, and act on signals conveying fitness-relevant information about others [1]. Difficult-to-fake signals are especially valuable to receivers because they generally convey honest information about the signaler’s condition [2,3]. A subset of these honest signals—sexually-selected, dimorphic signals—are thought to convey genetic-quality information to potential mates and competitors [4–6]. In humans, faces may serve as an especially rich site for such signals [7–11].

In recent years, facial width-to-height ratio (fWHR) has been proposed as one such sexually-selected signal in humans [12,13]. Proponents of this idea suggest that men’s fWHRs are developmentally mediated by testosterone (T), independent of body size [14]. Therefore, individuals sensitive to fWHR are able to make useful predictions about other traits associated with T, including strength, aggression, and dominance [15,16]. In support of this correlational premise, a number of studies have found associations between adult men’s fWHR and *perceived* likelihood of aggression, dominance, and prosocial behavior [17–22]; *actual* rates of aggressive and prosocial behaviors [23–27]; *success* in athletics, politics, and business [17,12,28–32]; and with *T itself* [16]. Furthermore, these behavioral traits (i.e., aggressive and other social behaviors) are linked to T [33–35] and are sexually dimorphic themselves [36,37], thus supporting the notion that fWHR may be affected by T as well, and hence provide useful predictive information about other T-associated traits.

In spite of these suggestive correlations, empirical evidence that fWHR is a signal of T and T-derived traits is mixed. Several studies—including one large-sample, multi-ethnicity study—found no evidence of sexual dimorphism in adult fWHR [38–40]. Other research has failed to show evidence for an association between adult fWHR and a number of known T-derived traits [40–42]. Several reasons have been proposed to account for the inconsistencies in research findings [39]: 1) Relatively small and university-based samples may have been subject to sampling biases (e.g. [12]). 2) Variation in fWHR across ethnic groups in the same sample may obscure sexual dimorphism in fWHR [39,43]. 3) The age- or sex-dependent presence of facial fat may conceal variation in craniofacial dimensions [38,44–45].

We evaluate whether fWHR is a sexually-selected signal by addressing these and other important gaps in the current literature. First, all human male secondary sexual characteristics, including, for example, masculinized voices and greater muscle mass [46–48], change dramatically during adolescence. Many of these changes are spurred by increases in endogenous T, beginning in early puberty [49,50]. Likewise, craniofacial growth is canalized during this time [14,43,51], changing little in response to subsequent variation in adult T levels. Therefore, T’s principal effect on fWHR necessarily occurs before adulthood, and fWHR ought to reflect T at the time of its development. We therefore examine the associations between fWHR, T-derived traits, and T itself during adolescence. By targeting an adolescent population, we also address a corollary issue in the study of facial masculinity: Masculinity is usually operationalized by dimensions that differ between adult males and females; thus, in the literature on facial shape, “masculinity” is often *de facto* defined in a way that is not only dependent on male facial growth trajectories, but also on female facial growth trajectories (e.g. [52,53]; cf. [54]). In the present study, we specifically target T-dependent male facial growth. We do so because the supposed signal value of “facial masculinity” is typically argued to rest on its positive correlation with T, not on its inverse correlation with the hormonal drivers of female facial growth. In other words, sexual dimorphism tautologically is defined by the phenotypes of *both* males and females and, therefore, cannot serve as a proxy for the hormone profile of only males (see also [55]).

A second insufficiency in the current literature is that it has focused primarily on wealthy Westernized populations (e.g. [12,14,17,23,40,42,56]). However, if fWHR is influenced by pubertal T, the causal relationship between the two ought to be cross-culturally observable. More importantly, because T compromises immune function [57], its developmental effects should be sensitive to surplus immune and energetic capacity [58,59]. The ontogenetic programs shaping facial growth almost certainly evolved in environments where energetic and immune stresses were significant, and studying their operation under similar circumstances provides superior ecological validity. Thus, we focus on adolescent Tsimane males, who belong to an indigenous population living in the Bolivian Amazon under energetically and immunologically stressful conditions.

Third, several studies have noted ethnic differences in facial bone structure [43], which may introduce bias in multi-ethnic samples [39]. The Tsimane represent a homogenous population, free from any potential confounds generated by inter-ethnic variation.

Fourth, several studies have shown that BMI is positively associated with fWHR [38,39,44], suggesting that facial fat may obscure variation in craniofacial dimensions and should be controlled in analyses. However, because BMI also confounds fat with muscle mass, we use an adolescent-specific adiposity algorithm in order to better control for differences in body fatness.

Finally, we include three other candidate facial-masculinity ratios in addition to fWHR that have been used in previous research: the ratio of facial width to lower face height (*fWHR-lower;* [39]), *cheekbone prominence* [39,52,60], and the ratio of the lower face height to full face height (*lower/full face height*; [39,52,60]). fWHR-lower and cheekbone prominence are smaller in adult men compared to women; whereas lower/full face height is larger among adult men as compared to women [39].

If fWHR, fWHR-lower, cheekbone prominence, and lower/full face height are honest indicators of pubertal T, several predictions should be supported:

1. Because male fWHR should develop during adolescence as T increases, fWHR should show a linear association with both age and T.
2. Like other secondary sexual characteristics (e.g., vocal fundamental and formant frequencies; [47]), fWHR should show evidence of a growth spurt, exhibiting two distinct features: a non-linear, sigmoidal growth pattern and a peak velocity, both linked with the developmental spurt in T.
3. fWHR should be positively correlated with other T-mediated traits during adolescence, such as upper-body strength and vocal fundamental frequency (referred to here as voice pitch).
4. The relationships between fWHR, T, and T-mediated traits (strength and voice pitch) should remain significant after controlling for age as the pace of maturation will vary among individuals of the same age.
5. These relationships should also survive controls for potential spurious variables, such as adiposity and height.

## Methods

To address these predictions, T, adiposity, upper-body strength, voice pitch, height, and age were measured in peri-adolescent subjects.

### Participants

Participants consisted of 91 peri-adolescent Tsimane males aged 8–23 (M = 13.8; SD = 3.5; see [47], Table 1, for N’s by age) as part of the Tsimane’ Health and Life History Project (final N after exclusions was 75, see Data Analysis, below). The Tsimane are an indigenous group living in the Amazonian lowlands of Bolivia and practice foraging-horticulture with relatively few calories derived from market sources [61–63]. Because of significant pathogen loads and a limited food supply, Tsimane ecology more closely resembles the environment to which humans are adapted than do Westernized environments [63–65]. Data were acquired with the assistance of an interpreter and a local assistant.

**Table 1:** Zero-order (upper right triangle), and partial correlations controlling for age (lower left triangle).

	fWHR	fWHR-lower	Cheek-bone Prom-inence	Lower/ Full Face Ratio	Age	T	Adiposity	Strength	Height	Voice Pitch
fWHR	-	.44**	-.08	-.04	-.03	.13	.18	.04	.04	-.12
fWHR-lower	.50**	-	.59**	-.63**	-.55**	-.52**	-.22^†^	-.63**	-.61**	.54***
Cheekbone Prominence	-.09	.52**	-	-.41**	-.33**	-.31**	-.25*	-.38**	-.38**	.31**
Lower/Full Face Ratio	-.03	-.43**	-.27*	-	.64**	.57**	.40**	.67**	.69**	-.57***
Age	-	-	-	-	-	.82**	.26*	.86**	.86**	-.78***
Testosterone	.28*	-.13	-.06	.09	-	-	.26*	.86**	.86**	-.76***
Adiposity	.20*	-.09	-.19	.32**	-	.33**	-	.43**	.39**	-.30**
Strength	.12	-.36**	-.18	.32**	-	.54**	.42**	-	.95**	-.84***
Height	.14	-.32**	-.19	.36**	-	.53**	.34**	.82**	-	-.83***
Voice pitch	-.24*	.20^†^	.08	-.17	-	-.36**	-.19	-.52***	-.50***	-
Mean	1.67	1.29	1.25	0.59	13.84	50.16	18.96	-	146.50	219.27
Standard deviation	0.08	0.07	0.09	0.03	3.48	38.16	6.44	-	15.93	50.16

Note. fWHR: facial width-to-height ratio, i.e. facial width/mid-face height; fWHR-lower: facial width/lower face height; lower/full face height: the ratio of the lower face height to the full face height; T: testosterone.

^†^≤0.10

*p<0.05

**p<0.01

***p<0.001.

### Ethics statement

All procedures for this study (including consent) were approved by the University of California, Santa Barbara Institutional Review Board (IRB). Procedures were also approved by the Tsimane Government (Gran Consejo Tsimane), village leaders, parents, and study participants. Because many Tsimane do not read or write, parent and participant consent was verbal. Consent was documented on a separate participant list.

Participants’ ages were estimated using participants’ stated birth date and calendar age, which were checked against the Tsimane Health and Life History census. When calendar age and birth date were in conflict (N = 8; conflicting age estimates differed by 2 years for 2 participants and 1 year or less for all other participants), the census age was used (see [47] and [62] for detailed age-assignment methods). Because the onset and length of adolescent development can vary widely across individuals and populations [46], and because T does not peak until early adulthood [66], a wide age range was used to capture the full developmental range of somatic and endocrine variation.

### Anthropometrics

Anthropometric measures were collected using standardized methods, whereby measurements were taken from the right side, repeated twice, and averaged for analysis [67,68]. Adiposity was measured with a Harpenden caliper by combining tricep, subscapular, and suprailiac skinfold thicknesses using Slaughter’s adolescent-specific algorithms [69]. Strength was calculated as a standardized average of handgrip strength (via pneumonic hand dynamometer) and as flexed bicep size (via tape measure): an experimentally validated proxy for overall strength [10,70,71].

### Acoustic measurement and analysis

Voice samples were recorded in mono using a Sony PCM-M10 digital audio recorder (44,100 Hz sampling rate and 16-bit quantization). A headset-mounted Audio-Technica lavalier microphone was positioned 5 cm from the mouth for each participant. For the recording, participants named each different object in five photographs. Recordings were saved as high-quality uncompressed linear PCM.wav files. Recordings were measured for mean fundamental frequency using Praat voice analysis software (version 5.1.37). Praat’s default settings were retained for all analyses. For additional detail see [25].

### Testosterone assays

Testosterone levels were assessed via 1-mL salivary samples passively drooled into polystyrene cryotubes. The saliva was relatively bubble-free, and to prevent contamination, participants washed their mouths prior to collection. To preserve the samples in transit, cryotubes were stored in liquid nitrogen, shipped to the University of California Santa Barbara on dry ice, kept frozen at -80 degrees C, and finally shipped on dry ice for analysis at Salimetrics LLC (State College, PA).

Although T typically has a diurnal rhythm in adult males, constraints on participants’ schedules meant that testing was conducted both in the morning and the afternoon. However, despite the range in testing times, T was not significantly associated with time of day (see [25] for further detail). Several factors may explain this. First, diurnal patterns do not emerge in adequately-nourished males until middle-adolescence [72]. Furthermore, males in populations with greater environmental stresses and delayed development might initiate diurnal cycles even later in life: research amongst the Ache—a Paraguayan group living in similar ecological conditions to the Tsimane—shows that peak AM:PM T ratios do not emerge until males are in their thirties [73]. Second, approximately 85% of the T samples were collected at least four hours after participants had awakened, by which time T would have stabilized even if it was diurnally rhythmic [72].

Assays were executed in duplicate by Salimetrics LLC using a sensitive competitive enzyme immunoassay protocol, and analytes were measured in picograms per milliliter. The average intra-assay and inter-assay coefficients of variation were 4.6% and 9.8% respectively. Salivary free T is strongly correlated with serum free and total T [74,75]; however, because salivary T is lower than serum T, the iron-binding glycoprotein transferrin was also assayed during analysis (M±SD = .91±0.89, range: 0.08–5.0) to adjust for potential blood contamination in participants’ saliva [76].

### Facial measurement

**Fig 1:**
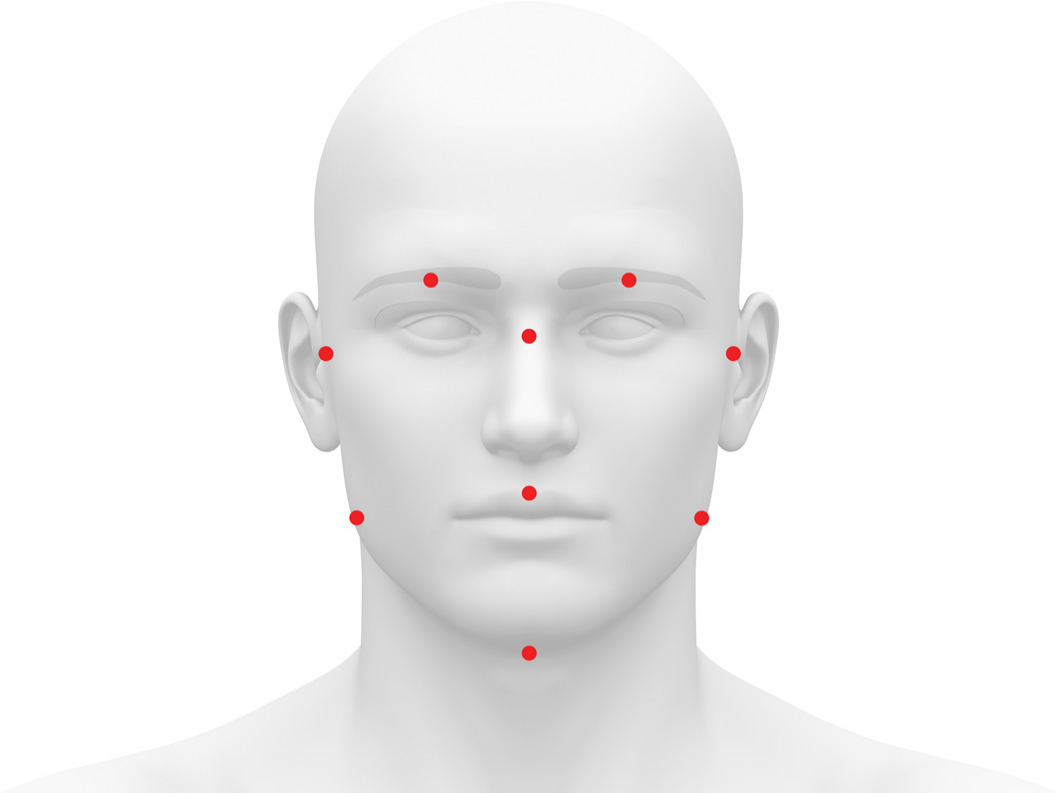
Facial landmarks used to derive facial masculinity ratios.

To obtain measures of fWHR, high-resolution, front-facing color photographs were taken with neutral expressions. The head was positioned in the medial-sagittal plane. Facial landmarks (e.g., lowest point of the chin along the facial edge) were marked on the photographs by three research assistants using the image-editing software GIMP (see Fig 1); the research assistants neither knew each other, the participants, nor were they aware of the hypotheses. Assistants recorded the x-y coordinates for each landmark twice, then all coordinates were averaged (in total, six x-coordinates, six y-coordinates per landmark) to establish the final landmark coordinates (Cronbach's alpha = .88). Facial measurements were calculated by inputting the endpoint coordinates into the Pythagorean Theorem (e.g., total face height comprises the total distance from the center of the hairline to the lowest point of the chin). The Pythagorean Theorem allowed us to accurately capture the full length of a feature by accounting for both its vertical and horizontal components, which is helpful when measuring features prone to fluctuating asymmetry (e.g., nose length) or features that do not lay perpendicular to the sagittal plane (e.g., eyes). In accordance with previous research (e.g., [77,78]), final feature measurements were standardized using inter-pupillary distance.

**Fig 2:**
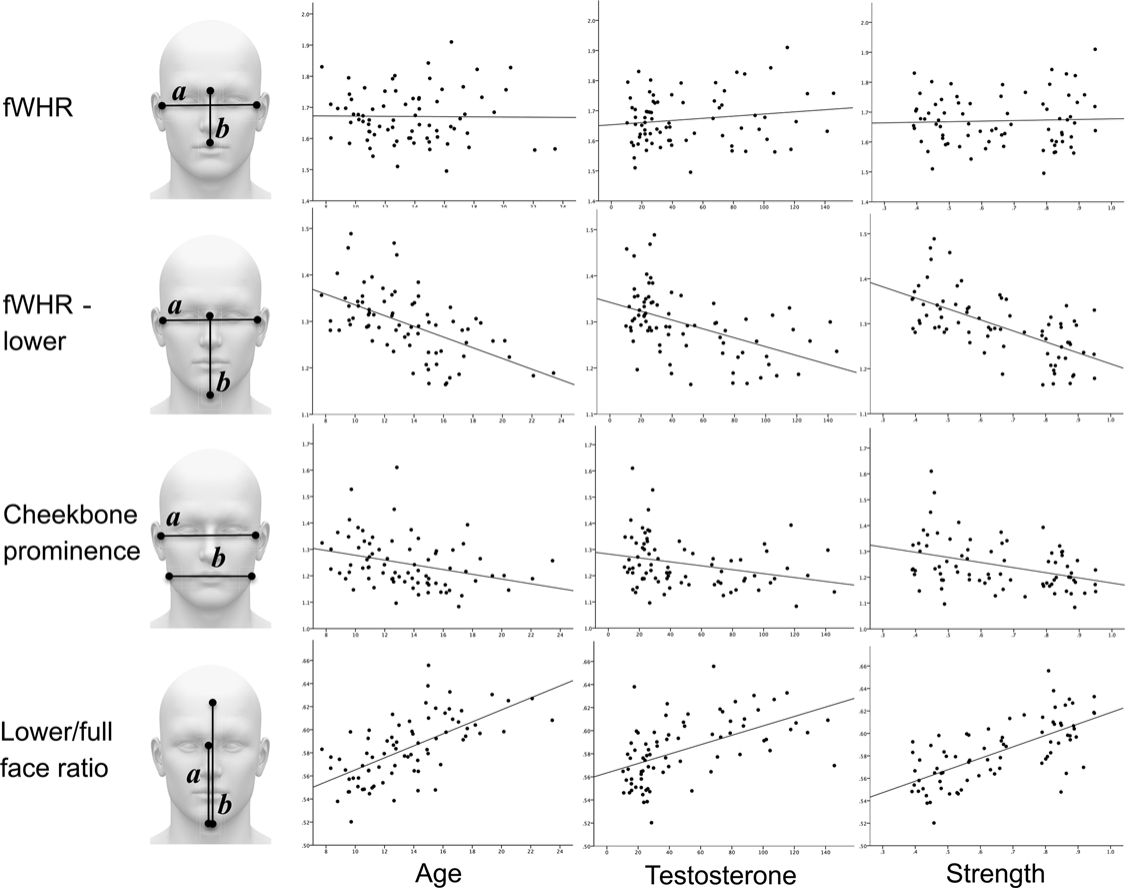
Facial masculinity ratios (*a/b*) by age, testosterone, and strength.

We calculated fWHR by replicating the method of Carré & McCormick [12]: bi-zygomatic breadth divided by the height of the face's midsection from the center of the face at the middle of the eye brows to the center of the upper lip (see Fig 1 for individual points and Fig 2 for facial ratios). Our three additional facial masculinity ratios are defined as follows: 1) facial width/lower face height (fWHR-lower) is the bi-zygomatic breadth divided by the height of the lower face (i.e. from the center of the face at the middle of the eye brows to the bottom of the chin; [39]); 2) cheekbone prominence is the bi-zygomatic breadth divided by the width of the face at the corners of the mouth [39,52,60]; and 3) lower/full face height is the height of the lower face divided by the full face height (the center of the hairline to the bottom of the chin; [39,52,60]).

### Data analysis

Participants were excluded from analysis for several reasons: 1) 6 participants’ saliva were discarded due to a damaged liquid nitrogen tank. 2) Outliers (with values greater than 3SDs from the mean) for transferrin (N = 3) and T (N = 1) were removed, 3) 6 participants’ lacked a photograph (due to a malfunctioning camera battery). The final N after exclusions was 75 (ages 8 to 23).

For regression analyses, T, height, strength, voice pitch, and age were log-transformed to match Pearson’s correlation assumption of normality. Variance inflation factors (VIFs) were used to examine multicollinearity in multiple regression, which were less than 6.0 for all models.

CurveExpert Professional software (version 2.2.0) was used to fit seven candidate non-linear, sigmoidal models to the pattern of age-related change in facial masculinity ratios in order to determine the presence of a growth spurt. Two measures were used to determine a best-fit model: Akaike Information Criterion-corrected (AICc; [79]) and the coefficient of determination (*R*^2^) from the linear model. AICc is used as the basis for model selection by identifying whether additional parameters (i.e., greater model complexity) are justified by the increase in model fit. In other words, AICc discourages overfitting. Lower AICc is preferred, and a general rule of thumb (with a sample size less than 256) for selecting between two models A and B is |AICc_A_−AICc_B_| > 6.0 [80].

## Results

The following analyses were designed to evaluate whether fWHR (and the three other facial masculinity ratios) are sexually-selected signals in human males.

(a) Does male fWHR (and other facial masculinity ratios) change with age and T during adolescence?

Zero-order correlations showed that fWHR is not positively associated with age (*r* = -0.03, *ns*) nor with T (*r* = 0.13, *ns*) during male adolescence. In contrast, all three other facial masculinity ratios—fWHR-lower, cheekbone prominence, and lower/full face ratio—revealed significant linear associations with age (*r* = -0.55, *p*<0.001; *r* = -0.33, *p*<0.01; *r* = 0.64, *p*<0.001, respectively), and with T (*r* = -0.52, *p*<0.001; *r* = -0.31, *p*<0.01; and *r* = 0.57, *p*<0.001, respectively). See Table 1 (upper right triangle) and Fig 2.

(b) Does male fWHR (and other facial masculinity ratios) show evidence of a growth spurt, in temporal contiguity with T’s developmental spurt?

**Fig 3:**
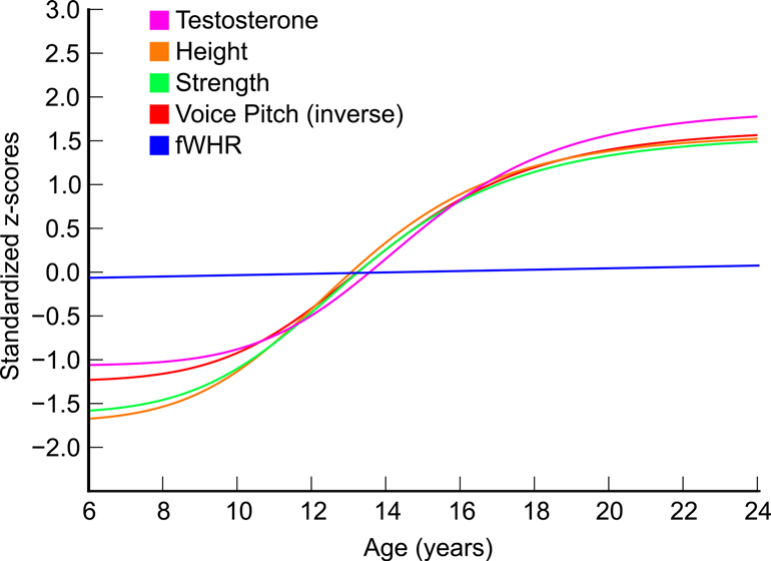
Lines of best fit for testosterone, strength, voice pitch, height and fWHR Note. Testosterone, strength, voice pitch, and height were best fit using non-linear sigmoidal models (see [47] for data on voice pitch and height). A linear model best described the data for fWHR.

First, T itself showed evidence of a developmental spurt: The best-fit sigmoidal model [Morgan-Mercer-Flodin (MMF); see [47]] demonstrates an AICc of 470.7 (*R*^2^ = 0.69), whereas the linear model is 483.7 (*R*^2^ = 0.62), for an AICc difference of 13. Using the MMF, the peak velocity for T occurs at age 14.2 in this sample (by comparison, peak velocity is 12.4 for height and 13.3 for voice pitch; see [47]). There was no difference in AICc between the linear model and the MMF (nor any of the other candidate sigmoidal models) for fWHR (-220.67 vs. -216.63), fWHR-lower (-473.79 vs. -470.35), cheekbone prominence (-413.73 vs. -411.19), and lower/full face ratio (-644.85 vs. -644.56). See Fig 3.

(c) Do male fWHR (and other facial masculinity ratios) correlate with other T-dependent traits, namely upper-body strength and voice pitch?

fWHR was not correlated with strength (*r* = 0.04, *ns*) nor with voice pitch (*r* = -0.12, *ns*) during adolescence, despite both of these dimorphic traits showing strong linear associations with T (Table 1). In contrast, fWHR-lower, cheekbone prominence, and lower/full face ratio all showed significant linear associations with strength (*r* = -0.63, *p*<0.001; *r* = -0.38, *p*<0.01; *r* = 0.67, *p*<0.001; respectively), and with voice pitch (*r* = 0.54, *p*<0.001; *r* = 0.31, *p*<0.01; *r* = -0.57, *p*<0.001; respectively). See Table 1 (upper right triangle) and Fig 2.

(d) Do the correlations between fWHR (and other facial masculinity ratios) and T, voice pitch, and strength remain after controlling for age?

Because we utilized a cross-sectional population, we control for age using partial correlations. The purpose of controlling for age in these analyses is strictly to draw out the developmental relationship between T and facial masculinity ratios. Results of these analyses showed that fWHR significantly correlates with T (*r* = 0.28, *p*<0.05) and voice pitch (*r* = -0.24, *p*<0.05), but not strength (*r* = 0.12, *ns*). After controlling for age, fWHR-lower was significantly associated with upper-body strength (*r* = -0.36, *p*<0.01) but was not associated with T (*r* = -0.13, *ns*) or with voice pitch (*r* = 0.20, *ns*). After controlling for age, cheekbone prominence was not associated with T (*r* = -0.06, *ns*), with upper-body strength (*r* = -0.18, *ns*), nor with voice pitch (*r* = 0.08, *ns*). Finally, after controlling for age, lower/full face ratio was significantly associated with upper-body strength (*r* = 0.32, *p*<0.01) but not T (*r* = 0.09, *ns*) nor voice pitch (*r* = -0.18, *ns*; Table 1, lower left triangle).

(e) Do fWHR (and other facial masculinity ratios) more closely correspond to T, adiposity, height, or age?

Multiple regression was used to discern whether facial masculinity ratios were better explained by T, adiposity, height, or age (Table 2). Results showed no significant predictors of fWHR or cheekbone prominence; however, height was a strong unique predictor of both fWHR-lower (β = -0.58, *p*<0.05) and lower/full face ratio (β = 0.59, *p*<0.01). In other words, adolescent males with long lower faces relative to both facial width and full face height are significantly taller (controlling for testosterone, adiposity, and age).

**Table 2:** Multiple regression models predicting facial masculinity ratios.

	Outcome variables			
Predictors	fWHR^a^	fWHR-lower^b^	Cheekbone prominence^c^	Lower/full face ratio^d^
Testosterone	0.43 (1.76^†^)	0.07 (0.34)	0.11 (0.46)	-0.24 (-1.36)
Adiposity	0.14 (1.05)	0.01 (0.09)	-0.15 (-1.19)	0.20 (2.11*)
Height	-0.05 (-0.17)	-0.58 (-2.59*)	-0.33 (-1.24)	0.59 (2.94**)
Age	-0.40 (-1.66^†^)	-0.11 (-0.57)	-0.09 (-0.39)	0.27 (1.55)

*Note*. Values represent standardized Betas (and standard errors in parentheses) from four separate multiple regression models. Predictors remain constant across all models, whereas the outcome variables differ.

^a^: *F*(4,73) = 1.77, *p* = 0.14. *R* = 0.31, Rsq = 0.09.

^b^: *F*(4,75) = 11.09, *p* < .001. *R* = 0.62, Rsq = 0.39.

^c^: *F*(4,75) = 3.22, *p* < .05. *R* = 0.39, Rsq = 0.15.

^d^: *F*(4,75) = 19.02, *p* < .001. *R* = 0.72, Rsq = 0.52.

^†^≤0.10

*p<0.05

**p<0.01.

## Discussion

Adolescence is a period of dramatic change in the male phenotype (e.g., voice, fat free mass, body shape), which is largely facilitated by sharp increases in T [47,49,66,72,81,82]. Based on several reports of sexual dimorphism in fWHR [14], a growing number of researchers have speculated that fWHR may be a sexually-selected signal mediated by pubertal T, which could honestly signal traits linked with this hormone [12,17,19–22,23–26,28,30–32,83]. In the present study, we found little support for this hypothesis in a sample of adolescent males: fWHR [12] was not correlated with age, T, upper-body strength, or voice pitch during male adolescent development. Further, fWHR showed no evidence of an adolescent growth spurt. This absence is striking because many secondary sexual characteristics experience dramatic changes in conjunction with the spurt in T (e.g., voice pitch; [47]; lean body mass; [81]; height; [82]). In contrast, three other facial masculinity ratios used in previous research (facial width/lower face height, cheekbone prominence, and lower face height/full face height; [39,52,60] were associated with age, T, upper-body strength, and voice pitch. Our results add to a growing literature that questions current suggestions about the signal content of fWHR [38–42,56].

This work expands previous research on fWHR in several important ways. First, it evaluates the association between facial masculinity ratios and T during the period of the lifespan when it is purported to have its effects: adolescence. If fWHR variation derives from T’s effect on craniofacial growth during puberty (Weston et al., 2007), three predictions should be supported: 1) fWHR should increase as T increases, 2) fWHR should reflect adolescent T (rather than adult T per se), and 3) fWHR should exhibit velocity changes during adolescence in parallel with the growth spurt in T. None of these effects were observed in this study. In addition, fWHR showed no evidence of a growth spurt at any time during adolescence. While the three other facial masculinity ratios were significantly associated with age and T, they also failed to show evidence of a growth spurt. Further, after controlling for adiposity and height, these alternative ratios were no longer associated with T. These findings stand in contrast to another plausibly sexually-selected male trait—voice pitch—which shows strong associations with T [49] and strength, even after controlling for potential confounds [48]. Thus, unlike voice pitch [48], these facial ratios do not seem to carry unique information above and beyond what may be observable from other aspects of phenotypic size.

Second, we explore the effect of T on male facial shape by examining adolescent male development rather than adult sexual dimorphism. T has important developmental influences on sexually-selected secondary sexual characteristics, and both T and its phenotypic targets (e.g., muscle mass, the vocal folds) exhibit considerable change during adolescence. By examining the changing phenotype in conjunction with changing hormones during male adolescence, researchers can directly assess whether or not particular traits are developmentally canalized by T and hence honestly reflect T levels during adolescence. Further, any sexually dimorphic measure conflates the nature and degree of male growth with female growth. In other words, attributing sexual dimorphism to T mistakenly assumes that the female face does not itself change during adolescent development (cf. [83–85]).

Third, we take an ecologically relevant approach by evaluating fWHR in a relatively homogenous, non-Western, under-developed population (cf. [56]). The Tsimane are potentially informative because they live under the types of high-pathogen, limited-food conditions where sexual selection would have shaped the form and ontogeny of various signaling systems.

Four, several researchers have noted that sex differences in facial adiposity may have led to the spurious conclusion that the fWHR is sexually dimorphic. A number of studies have shown that BMI is positively associated with fWHR in adults [38,39,49]; yet BMI confounds fat with muscle mass—which is itself subject to sexual selection pressures in males [70]. In the present study, we found no association between our measure of body adiposity and fWHR in adolescent males. We did, however, find associations between adiposity and the three alternative measures of facial masculinity. Further, after controlling for T, age, and height, adiposity remained a significant predictor of lower/full face ratio; that is, adolescents with more body fat have a longer lower face. These findings suggest that body fat is an important variable to consider in facial research using gestalt measurements that include soft-tissues.

We also explored a second potential spurious variable in the study of facial masculinity research: height. Both fWHR-lower and lower/full face ratio are ratios that depend on the size of the lower face (see Fig 2) relative to facial width or full-face height, respectively, and height was a significant predictor of these two ratios after controlling for T, age, and adiposity. This suggests that facial dimensions may grow in conjunction with increases in height (possibly because they are jointly influenced by somatotropic hormones like growth hormone; [86]) independently from T levels.

Finally, we explored three other facial masculinity ratios that have been examined in past research yet receive far less attention than fWHR [39,52,60]. Our results indicate that facial width/lower face height, cheekbone prominence, and lower face height/full face height are all significantly associated with age, T, strength, and voice pitch in this population. Specifically, facial width/lower face height gets smaller (i.e., the lower face grows more than the width of the face), cheekbone prominence gets smaller (i.e., the width of the face at the mouth—a measure of relative jaw width—grows more than the width of the cheekbones), and lower face height/full face height gets larger (i.e., facial growth is focused in the lower face) as male adolescents develop. These results are consistent with the craniofacial literature that documents pronounced growth in the male mandible under the influence of exogenous T [51] and during puberty [83–85]. Similarly, the association between T and these mandible-inclusive facial ratios accords with Lefevre et al. [16], who found significant sexual dimorphism in these three ratios, but no adult sex difference in fWHR. Although they showed no evidence of a pubertal growth spurt, overall findings suggest these facial ratios may be fruitful targets for future research.

In summary, this study adds to a growing fWHR literature that has been fraught with conflicting results. We have proposed several reasons that may account for these inconsistencies; however, more research is needed. One important avenue for future inquiry is the relationship between prenatal testosterone and masculine adult facial structure [87]. The ratio between the second and fourth digits (2D:4D)—which is sexually dimorphic [88] and associated with in-utero T [89,90]—is also associated with several measures of facial masculinity [87,91]. For instance, several studies have found a significant relationship between both adult and pre-pubertal fWHR and 2D:4D digit ratios, supporting this hypothesis [92,93]. 2D:4D is also associated with several behavioral traits, including aggression (e.g. [94]), that have been linked with fWHR [25]. Like 2D:4D, fWHR may be shaped by fetal T (cf. [87]), and the state of the current literature on fWHR may reflect that association. In the present research, fWHR showed no change during adolescence, no association with T or other known T-derived traits, nor evidence of a growth spurt; however, fWHR was significantly associated with T after controlling for age, adiposity, and height. It is unclear why an association was found under these narrow set of circumstances; however, it may derive from shared variance in prenatal and postnatal T. Similarly, 2D:4D changes little during puberty [95], but has shown inconsistent associations with postnatal T (for a review of the literature see [96]). Both prenatal and postnatal T are likely influenced by the same individual-specific physiology and genetic make-up (e.g. CAG repeats on the androgen receptor gene); therefore, this unexplained effect may derive from an association between prenatal and postnatal T, rather than a true causal link between pubertal T and fWHR. Future research, however, is needed to clarify this conjecture.

Furthermore, arguments about the signal value of fWHR must consider the ecological context and hence validity of the alleged message. What does it mean to say that the message only has content when the observer “controls for age”? Many developmental traits loosely co-vary with age and it is these traits, rather than age, that would have been the basis of inter-individual judgments. The signaler’s age is unlikely to have been an independent variable that observers could have used to adjust their perceptions in ancestral populations.

A recent article by Zebrowitz et al. [97] also challenges the alleged link between pubertal T and fWHR based on a body of research on “babyfaceness.” Babyfaced men (defined as having a rounder face, with relatively equal length and breadth, which is correlated with higher fWHR; [97]) show greater motivation, achievement, and aggressive behavior [98,99]. Zebrowitz et al. [97] suggest that the behavioral correlates of high fWHR (i.e. aggressiveness and assertiveness) may reflect compensatory responses by the babyfaced individual to perceptions that they are more naïve and warm than “maturefaced” peers. Zebrowitz et al. [97] also found that both babyfacedness and higher fWHR in adulthood were associated with a more uninhibited temperament in infancy, supporting the idea that early conditions likely impact both behavior and craniofacial growth.

There are several important limitations of the present study. First, we use a cross-sectional design, which offers limited control over individual differences; an optimal test of the presence of growth spurts would involve longitudinal data. However, other traits under the influence of sexual selection—like T, strength, and vocal frequencies [47]—show evidence of a growth spurt in cross-sectional data from this same population. Second, saliva samples were collected at variable times of the day. Although statistical analyses revealed no influence of collection time, ideal sampling for testosterone would institute a standard, morning collection time. Third, measurements were made on two-dimensional (2D) photographs. Although the majority of studies have measured fWHR in 2D photos (e.g. [12,17,24,41]), several researchers have argued that 3D images provide a more ecologically valid representation of human facial dimensions and should be utilized in future research (e.g. [87]). Fourth, although age estimates were based on two independent sources, actual birth date could not be validated using medical records; therefore, this work should be replicated in a population where birth certificates are available. Finally, the sample size is modest at 75; a larger number of participants might have revealed a relationship between T and fWHR, although this sample was sufficient to show a significant relationship between T and the three other measured facial ratios.

Overall, our results add to doubts about the status of fWHR as a sexually-selected signal for pubertal T and T-derived traits. Future studies should bring developmental data to bear in trying to reconstruct the effects of sexual selection. When using phenotypic traits as proxies for T dosage (for example when testing Red-Queen-based predictions, *sensu* [100]) it is important to target traits that are influenced by T and not merely sexually dimorphic.

## Supporting Information

S1 File fWHR data file. (SAV)
